# Helps to Hear

**Published:** 1882-12

**Authors:** 


HELPS TO HEAR. By James A. Campbell, M. D., Professor of Ophthalmology and Otology in the Homœopathic Medical College, of Missouri, etc., etc., with illustrations. Pp. 108. Price, 75 cts. Chicago: Duncan Brothers. 1882.

The object of this little work "is to offer to the profession and the general public a brief, practical review of the subject which is discussed," and the author has succeeded in so doing. Too little attention has been given this subject by the general practitioner, and in these 108 pages he will find much that will be of service to him and his deaf patients. The most important aids to hearing are well illustrated by cut and letter-press. We advise all to invest the small amount that is asked for it, with the assurance that they will find the money well spent. G. H. C.

